# BrkAutoDisplay: functional display of multiple exogenous proteins on the surface of *Escherichia coli* by using BrkA autotransporter

**DOI:** 10.1186/s12934-015-0316-3

**Published:** 2015-09-04

**Authors:** Fang Sun, Xiaoyun Pang, Tian Xie, Yujia Zhai, Ganggang Wang, Fei Sun

**Affiliations:** National Laboratory of Biomacromolecules, Institute of Biophysics, Chinese Academy of Sciences, 15 Datun Road, Beijing, 100101 China; Key Laboratory of Environmental and Applied Microbiology, Chengdu Institute of Biology, Chinese Academy of Sciences, Chengdu, 610041 China; University of the Chinese Academy of Sciences, Beijing, 100049 China

**Keywords:** Antibody display, Autotransporter, Bacterial surface display, Biocatalysts, BrkA

## Abstract

**Background:**

Bacterial surface display technique enables the exogenous proteins or polypeptides displayed on the bacterial surface, while maintaining their relatively independent spatial structures and biological activities. The technique makes recombinant bacteria possess the expectant functions, subsequently, directly used for many applications. Many proteins could be used to achieve bacterial surface display, among them, autotransporter, a member of the type V secretion system of gram-negative bacteria, has been extensively studied because of its modular structure and apparent simplicity. However, autotransporter has not been widely used at present due to lack of a convenient genetic vector system. With our recently characterized autotransporter BrkA (*Bordetella* serum-resistance killing protein A) from *Bordetella pertussis*, we are aiming to develop a new autotransporter-based surface display system for potential wide application.

**Results:**

Here, we construct a bacterial surface display system named as BrkAutoDisplay, based on the structure of autotransporter BrkA. BrkAutoDisplay is a convenient system to host exogenous genes. In our test, this system is good to efficiently display various proteins on the outer membrane surface of *Escherichia coli*, including green fluorescent protein (GFP), various enzymes and single chain antibody. Moreover, the displayed GFP possesses green fluorescence, the enzymes CotA, EstPc and PalA exhibit catalytic activity 0.12, 6.88 and 0.32 mU (per 5.2 × 10^8^ living bacteria cells) respectively, and the single chain antibody fragment (scFv) can bind with its antigen strongly. Finally, we showed that C41(DE3) is a good strain of *E. coli* for the successful functionality of BrkAutoDisplay.

**Conclusions:**

We designed a new bacterial display system called as BrkAutoDisplay and displayed various exogenous proteins on *E. coli* surface. Our results indicate that BrkAutoDisplay system is worthy of further study for industrial applications.

## Background

Recombinant exogenous proteins, polypeptides and antibodies displayed on the surface of phage, bacteria, fungi and virus, have wide applications, such as recombinant bacterial vaccines, peptide library screening, whole cell adsorption reagents, whole cell biocatalysts, whole cell solid reagents for clinical diagnosis and etc. [1–4]. In comparison to phage, fungi and virus, bacteria has been becoming a popular host for surface display application because of its simplicity of cell culture and genetic operation as well as its large capacity from small peptides to large proteins [5].

Bacterial inner membrane display system can successfully display exogenous proteins [6, 7]. However, it requires the preparation of spheroplasts since the exogenous proteins are displayed on the inner membrane and not freely accessible from the bacterial outer surface. In contrast, for bacterial outer membrane display system that exogenous proteins are displayed on the outer membrane directly, bacterial cells can maintain their integrity and viability during application. There are many advantages of bacterial outer membrane display system. First of all, laborious and costly purification steps are avoided. Instead, the whole bacteria are collected by centrifugation and applied directly for whole-cell biocatalyst, live vaccine screening and bioremediation [8, 9]. Secondly, the outer membrane display system can provide a genotype–phenotype link for protein libraries screening [10]. Furthermore, enzymes immobilized on the bacterial outer surface appear more stable in comparison with their free forms in solution [11].

There have been many proteins studied for displaying exogenous proteins on the bacterial outer membrane, including OmpA [12], flagellin, ice-nucleation protein (INP) [13] and autotransporters [14]. Among them, the surface display systems derived from autotransporters (ATs) of gram-negative bacteria exhibit great potentials for future wide application due to its apparent simplicity and modularity [9]. Most ATs typically act as adhesins, degradative enzymes, cytotoxins or other virulence factors [15, 16], including early discovered IgA protease from *Neisseria* [17], Ag43 from *E. coli* [18], AIDA from Enteropathogenic *E. coli* [19] and MisL from *Salmonella* [20]. All ATs contain an N-terminal cleavable signal peptide, a functional passenger domain and a C-terminal β-barrel domain [9]. The β-barrel domain of autotransporter is relatively uniform in size (~300 amino acids) and assembled on the outer membrane with the aid of the β-barrel assembly machinery (BAM) complex [21–24]. After the assembly of the β-barrel domain, the self-translocation of the passenger domain starts via the hairpin model that the C-terminal region firstly forms a hairpin in the central-pore of the β-barrel and the rest part of residues go through the pore by sliding from the C-terminus to N-terminus [25]. After the translocation of the passenger domain, the C-terminal residues left in the pore of the β-barrel reach a proper position and conformation ready for cleavage in the pore [26, 27]. Thus the β-barrel domain is, essential for passenger domain translocation across the outer membrane and is the key element for outer membrane display. ATs have been widely used as an elegant and efficient tool to display exogenous proteins and showed advantages for biotechnology and industrial (if scale up) applications [11]. However, not all target proteins can be successfully displayed [28] and till now there have been no universal rule to predict whether a target protein is suitable for AT-based surface display [9]. Furthermore, there is still lack of a convenient AT-based system for easier genetic operation and high throughput screening of target proteins.

The BrkA (*Bordetella* serum-resistance killing protein A) is an important virulence factor in *Bordetella pertussis* that confers serum resistance and mediates adherence [29]. We previously solved the crystal structure of the transmembrane β-domain of BrkA and demonstrated that the hairpin-like structure and the hydrophobic cavity at the periplasmic side of the β-domain are crucial for BrkA passenger domain translocation [30]. Besides, more interestingly, after the passenger domain is transported across the outer membrane and self-cleaved [27], the passenger domain of BrkA remains tightly associated with the bacterial surface [31]. This is a significant characteristic differing from other autotransporters. Previous extensive structural and functional studies of BrkA have enabled us to develop a BrkA-based bacterial display system, called as BrkAutoDisplay, by replacing the passenger domain region with a multi cloning site ready for various exogenous proteins insertion and secretion.

## Results

### Construction of a vector system for BrkAutoDisplay

**Fig. 1:**
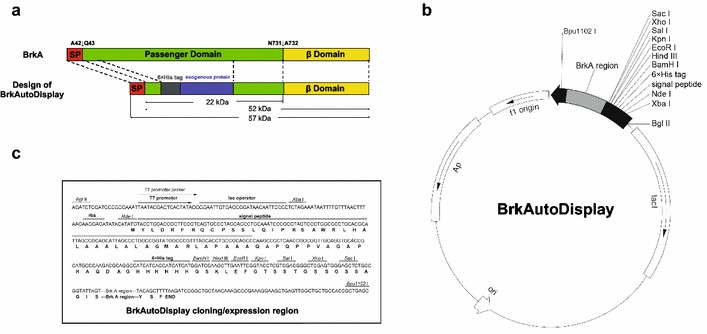
The construction of the vector BrkAutoDisplay. **a** BrkA domain organization and the design of BrkAutoDisplay. SP, the signal peptide. The signal peptide-processing site and the passenger domain cleavage site are indicated with *black arrows*. All other labels are self-explanatory. **b** The vector map of BrkAutoDisplay. **c** The multi-clone region of the vector BrkAutoDisplay

The BrkA protein is translated with an N terminal signal peptide followed by the passenger domain and its C terminal β-barrel domain (Fig. 1a). Based on our previous structural study of BrkA, we designed a new bacterial display system, which is called as BrkAutoDisplay, by replacing the passenger domain with other exogenous proteins (Fig. 1a). BrkAutoDisplay is a vector system derived from pET-22b (+) plasmid (Novagen) by inserting a synthesized DNA fragment between NdeI and Bpu1102I restriction sites (Fig. 1b). The DNA fragment (Fig. 1c) consists of the 5′ end nucleotides sequence encoding N terminal signal peptide (4-129nt), the sequence encoding a 6xHis tag (181–198nt), the multiple cloning site (MCS, 199–249nt), the sequence encoding the translocation determinants (250–810nt) [30] and the sequence encoding the β-barrel domain (811–1650nt). To be noted that, the sequence between the signal peptide and the 6xHis tag derives directly from the N-terminal residues of BrkA and the purpose of this design is to try to avoid any potential influence of the signal peptide cleavage efficiency.

The exogenous gene can be inserted into the MCS region by molecular cloning and then the recombinant plasmid can be transformed into *E. coli* for protein expression and surface display assay. With the signal peptide, the translated fusion exogenous protein will be transported into the periplasmic space via a Sec-dependent pathway. After the signal peptide is cleaved, the exogenous protein can translocate across the outer membrane under the help of the β-barrel domain. Due to the characteristic of BrkA protein, the final transported exogenous protein will adhere onto the bacterial surface [30]. By using the BrkAutoDisplay vector, it is convenient to construct a bacterial display system for different target proteins with a single step of molecular cloning.

Furthermore, the N-terminal fused 6xHis tag is designed to detect the expression level as well as the surface display efficiency of the target protein conveniently by western blot. Since the passenger domain of autotransporter is transported via the hairpin model that the C-terminal region firstly forms a hairpin in the central-pore of the β-barrel and the rest part of residues go through the pore by sliding from the C-terminus to N-terminus [25], its C-terminal residues firstly come out to the surface and thus the successful detection of the displayed N-terminal His-tag would indicate a complete secretion of the whole target protein.

Finally, the detected surface-displayed target protein would exhibit an apparent molecular weight of 22 kDa plus the theoretical molecular weight itself. For the un-cleaved portion, the detected apparent molecular weight would be 52 kDa plus the theoretical molecular weight of target protein (Fig. 1a).

### Displaying green fluorescent protein on the surface of *E. coli*

**Fig. 2:**
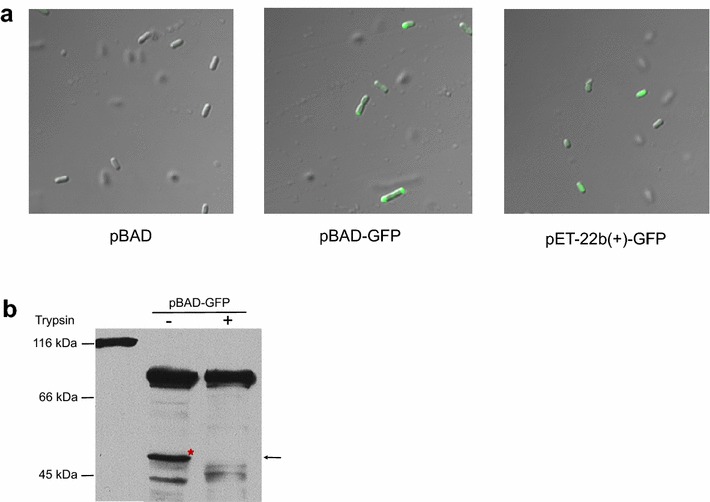
Surface display of GFP by BrkAutoDisplay. **a** Cells transformed with empty pBAD, pBAD-GFP and pET22b(+)-GFP were observed using fluorescence microscope. **b** Trypsin accessibility assay of the displayed GFP

We first tested the BrkAutoDisplay system by using the green fluorescent protein (GFP, 27 kDa). GFP gene was cloned into BrkAutoDisplay vector and the recombinant plasmid was named as pBAD-GFP. The pBAD-GFP was transformed into *E. coli* C41(DE3), to obtain the recombinant bacteria for GFP surface display. At the same time, the empty BrkAutoDisplay vector, pBAD, was transformed into the same strain of *E. coli* as a control. The expression and surface display of GFP was assessed by fluorescence microscopy and trypsin accessibility [30]. For the experimental group, significant green fluorescence signals appear at the ends of rod-shaped bacteria (Fig. 2a), indicating a successful expression of GFP in its natural fold. Furthermore, the localization of GFP suggested that the BrkAutoDisplay system could display the target protein on the surface with a specific spatial distribution. As a control, although the cells transformed with pET-22b(+)-GFP plasmid could also express GFP successfully, green fluorescence signals appear over the whole body of the bacteria, showing no preferred spatial distribution (Fig. 2a). To be noted, BrkA has been reported to contain a sequence determinant for its localization on the pole of *E. coli*, which is relevant to the LPS (lipopolysaccharide) and membrane fluidity of *E. coli* outer membrane [32].

To further verify whether the expressed GFP has been displayed on the surface, we performed trypsin accessibility assay (see “Methods”). As shown in Fig. 2b, for pBAD-GFP construct without trypsin treatment, both the 79 kDa full-length un-cleaved protein and the 49 kDa cleaved transported GFP can be detected by an anti-His antibody. Treating the whole cells with trypsin resulted in the complete digestion of the 49 kDa moiety, indicating that the transported GFP has been successfully displayed on the surface of the cell. Because it will normally take several minutes for autotransporter to complete the passenger secretion process [33], the 79 kDa full-length protein represents the intermediates inaccessible to trypsin, which is located in the periplasm or is only targeted to the OM without passenger translocation.

### Displaying catalytic enzymes on the surface of *E. coli*

The second round of test to assess the potential application of BrkAutoDisplay for whole cell biocatalysts. We selected six different enzymes to verify their surface display abilities by BrkAutoDisplay since their cDNAs are accessible, which include CotA from *Bacillus subtilis* (GenBank No. AIC43241.1), ECH from *Caenorhabditis elegans* (GenBank No. NP_491222.2), ECH-9 from *Caenorhabditis elegans* (GenBank No. NM_069474. 4), AtaPT from *Aspergillus terreus* (GenBank No. KP893683), EstPc from *Psychrobacter cryohalolentis* K5^T^ (GenBank No. CP000323) and PalA from *Pseudomonas sp* (GenBank No. AY055203). CotA (MW 58 kDa) is a classical laccase, catalyzing the reduction of oxygen to water accompanied by the oxidation of polyphenol compounds. The laccase has been widely applied into textile industry, food engineering, medicine in organic synthesis and bio-remediation of contaminated environment [34]. ECH and ECH-9 are key enzymes for the cellular β-oxidation cycle and responsible for the second and third steps of reactions respectively [35, 36]. The ECH (MW 29 kDa) is an enoyl-CoA hydratase and adds a hydroxyl group and a proton to the unsaturated β-carbon of a fatty-acyl CoA and the ECH-9 (MW 49 kDa) is a L-3-hydroxyacyl-CoA dehydrogenase and oxidizes the hydroxyl group of L-3-hydroxyacyl-CoA to a keto group. AtaPT (MW 46 kDa) is an aromatic prenyltransferase and responsible for the biosynthesis of diverse active prenylated aromatics [37]. The EstPc (MW 33 kDa) is a cold-active esterase that exhibits high activity at low temperature (15–25 °C) to catalytic hydrolysis of p-nitrophenyl butyrate [38]. The PalA (MW 38 kDa) is a lipolytic enzyme that hydrolyzes triglycerides into glycerol and free fatty acids [39].

**Fig. 3:**
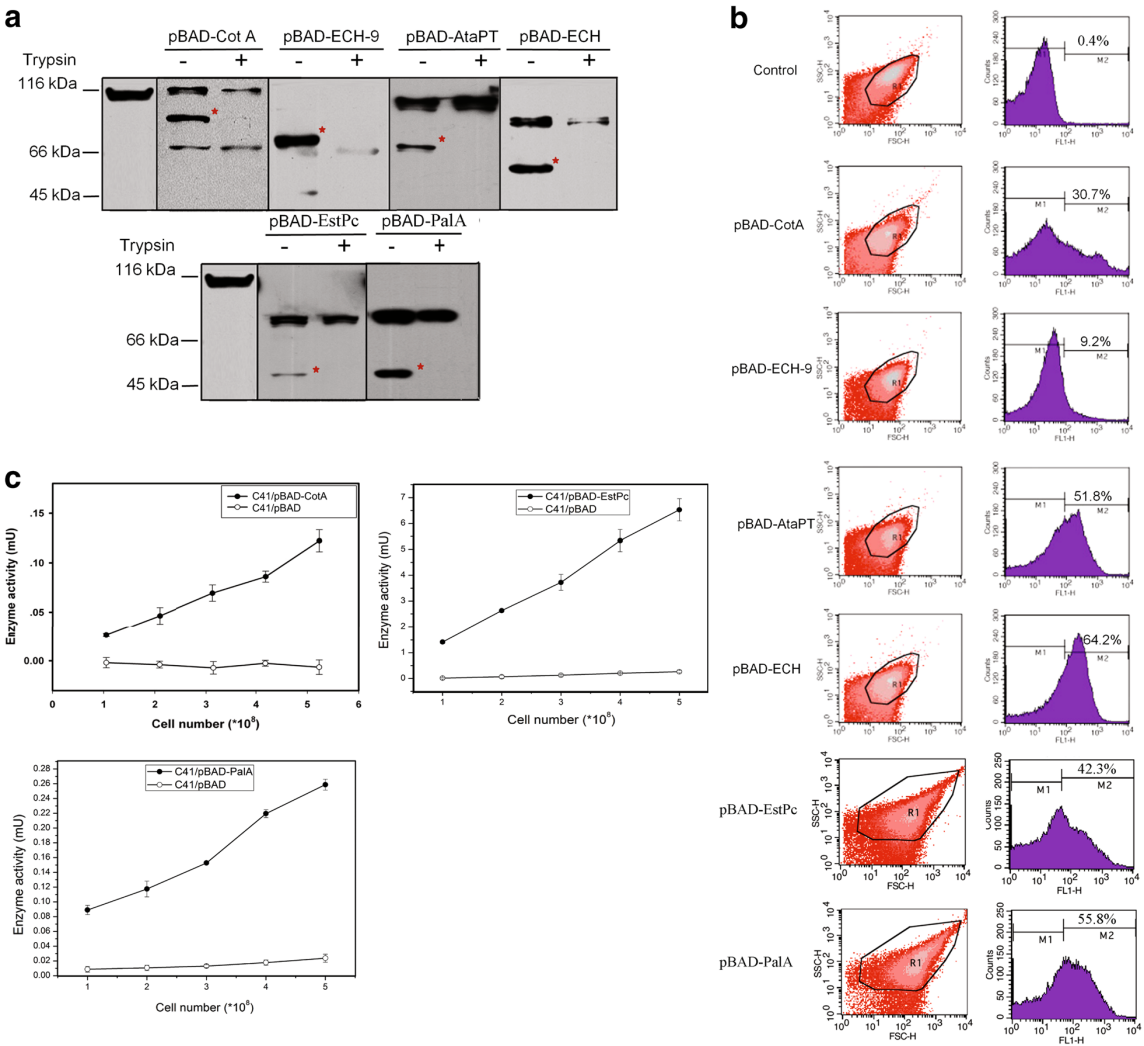
Surface display of enzymes. **a** Trypsin accessibility assay of the displayed enzymes. **b** Surface display efficiency quantification by flow cytometry. **c** Enzymatic activity assays of the cells with displayed enzymes (CotA, EstPc and PalA) and the control group with empty pBAD. The enzymatic activities were calculated and calibrated (see “Methods”)

The DNAs of CotA, ECH-9, AtaPT, ECH, EstPc and PalA were inserted into BrkAutoDisplay vector and then transformed into *E. coli* C41(DE3) to get six constructs, pBAD-CotA, pBAD-ECH-9, pBAD- AtaPT,pBAD-ECH, pBAD-EstPc and pBAD-PalA respectively. The expected molecular weights of the displayed targets are 81, 71, 68, 51, 55 and 60 kDa respectively. Treating the whole cells with trypsin resulted in the complete digestion of the corresponding moieties, indicating that all the six enzymes have been successfully displayed on the surface of the cell (Fig. 3a).

Beside trypsin accessibility assay, we further used flow cytometry to quantify the display efficiencies of those six enzymes (Fig. 3b). All the cells were treated with the anti-His antibody followed by FITC conjunct goat anti-mouse secondary antibody. Subsequently, the samples were investigated by flow cytometer. Only the successfully displayed enzyme with 6x-His tag in N-terminus could be labeled by FITC. As a result, the quantified display efficiencies on the surface of *E. coli* for pBAD-CotA, pBAD-ECH-9, pBAD-AtaPT, pBAD-ECH, pBAD-EstPc and pBAD-PalA are 30.7, 9.2, 51.8, 64.2, 42.3 and 55.8 % respectively, in comparison to the background value of 0.4 % for the control group (Fig. 3b). Both trypsin accessibility assay and flow cytometry data suggest successful displays of all six enzymes although variations of display efficiencies exist.

With the catalytic assay systems available for CotA, EstPc and PalA, we tested the catalytic activities of these displayed enzymes to further verify the ability of BrkAutoDisplay for the application of whole cell biocatalysts. As shown in Fig. 3c, the recombinant pBAD-CotA cells possess the laccase activity by oxidizing the substrates 2,2′-Azino-bis (3-ethylbenzothiazoline-6-sulfonic acid) diammonium salt (ABTS). The whole cell enzyme activity increases with the increase of the number of cells, and per 5.2 × 10^8^ bacteria cells can reach the activity of 0.12 mU CotA. As a control, the cells that were transformed with empty BrkAutoDisplay plasmid showed no laccase activity (Fig. 3c).

For the enzymatic activity assays of the recombinant pBAD-EstPc and pBAD-PalA cells, experiments were conducted at 25 °C by using p-nitrophenyl butyrate (C4) as the substrate [38]. The whole cell enzyme activity increases with the increase of the number of cells, and per 5.2 × 10^8^ bacteria cells can reach the activity of 6.88 and 0.32 mU for EstPc and PalA respectively. As a control, the cells transformed with empty BrkAutoDisplay plasmid showed no enzymatic activity (Fig. 3c).

As a result, the three kinds of bacteria here harvesting the corresponding recombinant constructs show significant catalytic activities, which can be improved for the future industrial applications.

### Displaying single chain antibody and comparison with Ag43 AutoDisplay system

Another important application of surface display is antibody presentation [40]. Here we selected a single chain antibody, ATscFv (MW 28 kDa), to assess the ability of BrkAutoDisplay to display an active antibody. ATscFv was cloned from Prof. Jie Tang’s lab (IBP, CAS) and it can recognize the antigen human tumor necrosis factor alpha (hTNFα). Xiao et al. has used the Ag43-based AutoDisplay system to display the active ATscFv successfully by comparing with the INP-based surface display system [41]. In the present study, we will further compare the display abilities and efficiencies of ATscFv between Ag43-based system (Ag43β) and our BrkAutoDisplay system.

**Fig. 4:**
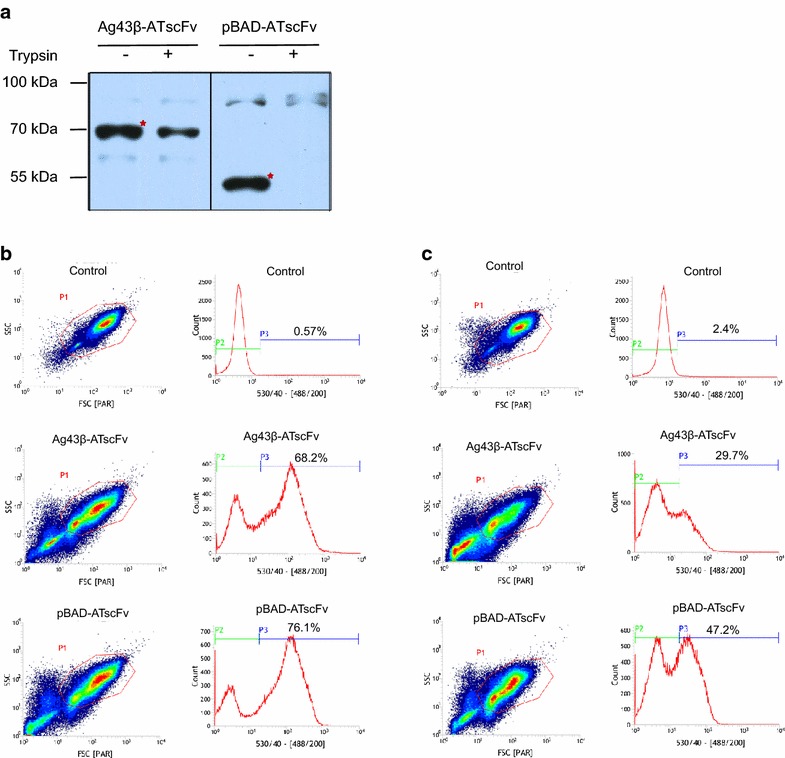
Surface display of ATscFv and comparison with Ag43-based surface display system. **a** Trypsin accessibility assay of the displayed ATscFv. **b** Surface display efficiency quantification by flow cytometry. **c** Antigen binding ability of displayed ATscFv quantified via flow cytometry

The ATscFv gene was inserted into the BrkAutoDisplay plasmid with the name of pBAD-ATscFv. Then the recombinant plasmids, pBAD-ATscFv and Ag43β-ATscFv (a gift from Prof. Hai-Ying Hang), were transformed to *E. coli* C41(DE3). The expected molecular weights of the displayed ATscFv are 50 and 64 kDa, respectively. Trypsin accessibility assay revealed that ATscFv can be significantly displayed on the bacterial surface for pBAD-ATscFv while less efficient for Ag43β-ATscFv (Fig. 4a).

The display efficiencies of pBAD-ATscFv and Ag43β-ATscFv were further quantified via cell cytometry approach. All the cells were treated with the anti-His antibody followed by FITC conjunct goat anti-mouse secondary antibody and then investigated by flow cytometer. Only the successfully displayed enzyme with its N-terminal 6x-His tag could be labeled by FITC. As a result, the quantified display efficiencies of ATscFv for pBAD-ATscFv and Ag43β-ATscFv are 76.1 and 68.2 %, respectively, in comparison to the background value of 0.57 % for the control group (Fig. 4b).

To detect antigen-binding ability of displayed AtscFv, the liquid cells were incubated with the antigen GFP-hTNFα and thereafter examined via flow cytometry (Fig. 4c). As a result, the quantified antigen-binding efficiencies for the cells of pBAD-ATscFv and Ag43β-ATscFv are 47.2 and 29.7 %, respectively, compared with the background value of 2.4 % (Fig. 4c). The less antigen-binding efficiency (Fig. 4c) in comparison to the surface display efficiency (Fig. 4b) suggests that there are portions of displayed ATscFv lack of antigen binding ability, which might be due to the failure of correct folding, the potential spatial obstacle or other unknown reasons.

All of above, both Ag43-based system and BrkAutoDisplay system can successfully display single chain antibody ATscFv on the surface of the cell with significant antigen binding activity, while, the current data suggested that BrkAutoDisplay system played a better performance than Ag43-based system in either antibody display efficiency or antigen binding activity.

### Display abilities of BrkAutodisplay by different bacterial strains

**Fig. 5:**
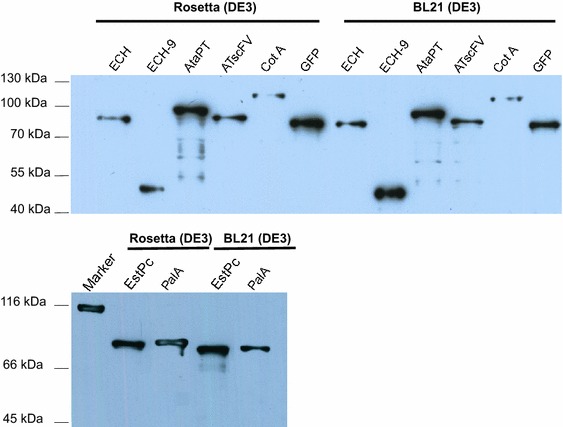
A western blot showing the failure of surface display by BrkAutoDisplay for the strains *E.coli* BL21 (DE3) and Rosetta (DE3)

The above bacterial surface display assays were performed by using the strain of *E. coli* C41 (DE3). To investigate whether the exogenous protein display by BrkAutoDisplay system is strain-dependent, we transformed the above recombinant plasmids into different strains of *E. coli* Bl21 (DE3) and *E. coli* Rosetta (DE3) and assessed the surface display abilities of those trains. After induction of the cells, the target protein expression levels were examined by western blot using anti-His antibody (Fig. 5). However, only the full-length un-cleaved moieties were observed for GFP, enzymes and ATscFv, suggesting a failure of passenger translocation onto the outer membrane [30]. Therefore, BrkAutoDisplay system is strain-dependent and does not work with the strain of *E. coli* Bl21 (DE3) and Rosetta (DE3).

## Discussions

In the present work, we designed a new autotransporter mediated bacterial surface display system BrkAutoDisplay based on the structure of BrkA. The system includes an easy-to-use plasmid with a multi-clone site and a workflow of assessing the surface display ability and efficiency, which enables other researchers to use this system easily via simple genetic operation and detect the efficiency of surface display via standard antibody and routine western blot assay. The unique characteristic of BrkA is that the cleaved passenger domain remains adhered on the bacterial surface, which is conducive to keep the displayed exogenous proteins associated onto the cell, thus, the functionalized bacteria can be recycled/re-used conveniently. Three kinds of exogenous proteins including GFP, enzymes and antibodies have been tested using this new bacterial surface display system, showing its advantages of the wide applicability, large capacity and high efficiency.

The successful display of GFP by BrkAutoDisplay system suggests that this system is applicable to display those proteins with β-barrel structural motif. Furthermore, in the future, the gene of GFP could be incorporated into the BrkAutoDisplay plasmid to enable the surface display of a GFP-fused target protein, which will make the assessment of the display ability and efficiency even easier via fluorescence signal and fluorescence microscopy.

Enzymes immobilized on surfaces appeared to be more stable compared to free molecules. Using BrkAutoDisplay system, it is possible by a standard protocol to decorate bacterial cell surfaces with target enzymes and produce large amount of these cells with a minimum of costs and equipment. Moreover, these cells can be recovered and reused in several subsequent process cycles [11]. The *E. coli* cells decorated with CotA, EstPc and PalA via BrkAutoDisplay system showed the significant laccase, esterase and lipolytic activities (0.12, 6.88 and 0.32 mUs per 5.2 × 10^8^ living bacterial cells) respectively, which would have wide applications in industry and environment bioremediation.

Moreover, for some enzymes, their activities need the presence of their chaperones. To display those enzymes on bacterial surfaces in their functional state, it is possible to co-express and co-display both the enzyme and its chaperone on the surface, e.g. the co-expression of the lipase and its chaperone foldase [2]. For this purpose, BrkAutoDisplay system can be further adapted to yield two plasmids containing different antibiotics genes and different copy operons, enabling co-expression of different target proteins.

Recombinant antibodies are increasingly used in many applications such as clinical diagnosis and therapeutics [40], which require antibodies with high antigen affinity and specificity [4]. Antibody surface display technology is critical for novel antibody screening and antibody affinity maturation. In the previous researches, Ag43β has been applied to present different vaccine candidates [9, 42, 43]. In the present work using the antibody of ATscFv, we found that BrkAutoDisplay has a superior performance than Ag43β, suggesting a potential application of BrkAutoDisplay for the future antibody development.

In the present work, we tested the strain-dependent display ability of BrkAutoDisplay found that only *E. coli* C41 (DE3) can work well in comparison to *E. coli* BL21 (DE3) and Rosetta (DE3). Since the overexpression of exogenous gene is normally toxic to the host, *E. coli* C41 (DE3) might have a better control of expression level of exogenous gene, which has enabled this strain to express many toxic membrane proteins, and might have additional mechanisms to help exogenous autotransporters to target onto the outer membrane, which is essential for the surface display of the exogenous proteins.

## Conclusion

BrkAutoDisplay is a new bacterial surface display system, which includes an easy-to-use plasmid with a multi-clone site and a workflow of assessing the surface display ability and efficiency. BrkAutoDisplay enables researchers to use this system easily via simple genetic operation and detect the efficiency of surface display via standard antibody and routine western blot assay. BrkAutoDisplay exhibits potencies of wide applications for whole-cell biocatalysts, environment remediation and antibody development. In the future, more work, such as additional testimonies using industrial enzymes and medical antibodies, stability test of the displayed target and scalable ability test of the system, will be performed.

## Methods

### Construction of the vector BrkAutoDisplay

The vector BrkAutoDisplay is derived from the vector pET-22b(+) (Merck Novagen) by inserting a synthesized DNA fragment (see RESULT) between the restrict digestion sites NdeI and Bpu1102 I. The DNA fragment was synthesized via the service from GeneWiz. The nucleotides from 130 to 180 of the fragment encode a peptide sequence corresponding the residues of BrkA from Glu^43^ to Gly^59^. The nucleotides from 250 to 1650 of the fragment encode a peptide sequence corresponding the residues of BrkA from Ala^545^ to Phe^1013^.

### Molecular cloning and surface display

The genes encoding GFP, CotA, ECH-9, AtaPT, ECH, EstPc, PalA and ATscFv were amplified by polymerase chain reaction (PCR) using the primer set in Table 1. The genes of ECH-9 and ECH were amplified from the cDNA library of *C. Elegans.* The gene of ATscFv is a kind gift from Prof. Hai-Ying HANG (Institute of Biophysics, Chinese Academy of Sciences, Beijing). The gene of AtaPT is a kind gift from Prof. Jungui Dai (Institute of Materia-Medica, Peking Union Medical College and Chinese Academy of Medical Sciences). The genes of EstPc and PalA were synthesized via the service from GeneWiz. All of the PCR amplified fragments were digested with the restriction endonucleases BamH I (or EcoRI) and Kpn I (or XhoI) and then linked to the BrkAutoDisplay plasmid that was pre-treated with BamH I (or EcoRI) and Kpn I (or XhoI), yielding the recombinant plasmids, pBAD-GFP, pBAD-CotA, pBAD-ECH-9, pBAD-AtaPT, pBAD-ECH, pBAD-EstPc, pBAD-PalA and pBAD-ATscFv. The recombinant plasmids were verified by sequencing, and then were transformed to *E. coli* strains C41 (DE3), BL21 (DE3) and Rosetta (DE3), respectively for protein expression. The empty BrkAutoDisplay plasmid was also transformed as a control. In addition, the recombinant plasmid ATscFv-Ag43β in pET-22b (+), a kind gift from Prof. Hai-Ying HANG, was transformed to *E. coli* strains C41 (DE3). The transformed cells were grown at 37 °C in Lysogeny Broth (LB) medium supplemented with ampicillin (100 μg/ml) until the OD_600_ reached approximately 0.8–1.0. Then the recombinant protein expression was induced by the addition of 0.2 mM IPTG (isopropyl β-d-1-thiogalactopyranoside) for 16 h at 16 °C.

**Table 1:** Primers for the target proteins cloning

Primer name	Sequence	Target protein to be displayed	Restriction sites
F1	5′-CGGGATCCATGGTGAGCAAGGGCGAG-3′	GFP	BamHI and XhoI
R1	5′-CCGCTCGAGCCCTTGTACAGCTCGTCCA-3′		
F2	5′-CGGGATCCATGACACTTGAAAAATTTGTGG-′	CotA	BamHI and XhoI
R2	5′-CCGCTCGAGCCTTTATGGGGATCAGTTA-3′		
F3	5′-CGGGATCCATGGGCAAAGTTCCAGAAGAAG-3′	ECH-9	BamHI and KpnI
R3	5′-GGGGTACCTAGTTTTGATGACATCAGTTTG-3′		
F4	5′-CGGGATCCCGGCCCTGGCAGATCCTGA-3′	AtaPT	BamHI and KpnI
R4	5′-GGGGTACCCACACGTGCGACATTTCCCGCAA-3′		
F5	5′-CGGGATCCATGTCTGGAAAAGTGGTTAG-3′	ECH	BamHI and KpnI
R5	5′-GGGGTACCTTTCTTGACGACCGCTTCGAG-3′		
F6	5′-CGGAATTCATAAATACCACCCA AAAGATTATTC-3′	EstPc	EcoRI and KpnI
R6	5′-GGGGTACCGTTCTTTAACCCTTCACGAAACGC-3′		
F7	5′-CGGAATTCATGATCAAAC AGACGTTGTTTGTACC-3′	PalA	EcoRI and KpnI
R7	5′-GGGGTACCTTCGTCCTGA TGAGCGCGCA ACGT-3′		
F8	5′-CGGAATTCATGGATATCGGAATTAATTCGGATCC-3′	ATscFv	EcoRI and XhoI
R8	5′-CCGCTCGAGCCCCGTTTTATTTCCAACTTTG-3′		

### Trypsin accessibility assay and western blot analysis

After induction for 16 h, 1 ml of culture was harvested by centrifugation at 2000*g*, 4 °C for 10 min and re-suspended in 200 µl of PBS. For 200 µl of cells, 4 µl of 10 mg/ml trypsin (Merck, 108367) was added. After incubation at 37 °C for 10 min, the cells were put on ice immediately and 20 µl of FBS (fetal bovine serum) was added to stop digestion. The cells were harvested, washed three times with PBS containing 10 % FBS and once in PBS alone. The washed cells were finally re-suspended in 5 % SDS loading buffer and boiled for 20 min. As a control, another 200 µl aliquot of cells was simultaneously processed in the same manner without added trypsin. Samples were resolved by SDS/PAGE (12 % gels) and transferred on to nitrocellulose membranes (Millipore) for 70 min at 300 mA at 4 °C. The non-secreted and secreted proteins were detected by anti-His antibody (Sigma) and horseradish peroxidase-conjugated goat anti-mouse secondary antibody (ZSGB-Bio) with a working dilution of 1:3000 and 1:5000 respectively. The membranes were incubated in luminal reagent (Millipore) and exposed to film.

### Fluorescence assay

The 200 μl culture was harvested and washed with 200 µl of PBS. Then 7 μl of samples were dipped onto a microscope slide, which was observed by confocal laser scanning microscope OLYMPUS FV1000.

### Assay with flow cytometry

The cells were incubated with anti-His antibody (1:3000 in PBS containing 1 % BSA) for 1 h at room temperature. Then the cells were washed again with PBS containing 1 % BSA, and incubated with FITC conjunct goat anti-mouse antibody (Thermo Scientific, Immuno Research, 1:100 in PBS containing 1 % BSA) for 1 h at room temperature in dark. After labeling, cells were washed once with PBS containing 1 % BSA and re-suspended in 1 ml of PBS containing 1 % BSA. The fluorescence associated with the cells were detected using FACS Influx cell sorter (BD Biosciences).

To detect the antigen-binding ability of the displayed ATscFv, the 1 ml culture was harvested and washed with PBS. Then the cells were incubated with GFP-hTNFα (2 mg/L in PBS containing 1 % BSA) for 1 h at room temperature in dark. After labeling, cells were washed once with PBS containing 1 % BSA and re-suspended in 1 ml of PBS containing 1 % BSA. The fluorescence associated with the cells was detected and sorted via FACS Influx cell sorter (BD Biosciences).

### Laccase activity assay of displayed CotA

The laccase activity was detected by monitoring the oxidation of 2,2′-Azino-bis (3-ethylbenzothiazoline-6-sulfonic acid) diammonium salt (ABTS, Sigma). The unit (1U) of laccase activity is defined as the amount of enzyme required for converting 1.0 µmol substrates in a minute under the condition of 40 °C and pH 5.0.

The culture was harvested and washed with the same step above. 50 µl of bacteria suspension, 900 µl of sodium citrate-Sodium dihydrogen phosphate buffer (pH5. 0, 100 mM) and 50 µl of ABTS (1 mM) aqueous solution were mixed. The mixture was incubated in 40 °C water for 3 min. The optical absorbance at 420 nm was monitored. The laccase activity can be calculated using the formula,

$$ \frac{{\varDelta OD_{420} \times V \times 1000}}{{{\text{t}} \times \varepsilon_{ABTS} \times {\text{d}}}} $$

where, $\varDelta OD_{420}$ is the change of the optical absorbance at 420 nm, t represents the reaction time (min), V is the volume of the reaction system (ml), $\varepsilon_{ABTS}$ is the molar absorption coefficient of ABTS at 420 nm (36 µmol/ml × cm) and d is the optical path (cm).

### Esterase activity assay of displayed EstPc

Esterase activity assays were performed using p-nitrophenyl butyrate (C4) as an ester substrate (Sigma). The reaction mixture contained 5 μl of a substrate (10 mM ester in 2-propanol), 190 μl buffer (20 mM Tris–HCl, 0.1 M NaCl, pH 9.0) and 5 μl of PBS washed intact cells. The absorbance at 415 nm was determined at 25 °C for 30 min with Multiskan (Thermo). One unit of activity was defined as the release of 1 μmol of p-nitrophenol per minute [38]. The esterase activity can be calculated using the following formula,

$$ \frac{{\varDelta OD_{415} \times V \times 1000}}{{{\text{t}} \times \varepsilon_{{4 - {\text{pnitrophenyl}}}} \times {\text{d}}}} $$

where, $\varepsilon_{{{\text{p}} - {\text{nitrophenyl}}}}$ is the molar absorption coefficient of p-nitrophenol at 415 nm (18 µmol/ml × cm).

### Lipolytic activity assay of displayed PalA

For the lipolytic activity assay of PalA, 5 μl of 10 mM p-nitrophenyl butyrate (Sigma) was added to 195 μl of PBS (pH 7.4) with re-suspended cultured cells. The absorbance at 415 nm was determined at 25 °C for 30 min with Multiskan (Thermo). The lipolytic activity of PalA can be calculated in a same way with EstPc above.

## Abbreviations

ABTS: 2,2′-Azino-bis (3-ethylbenzothiazoline-6-sulfonic acid) diammonium salt
Ag43: antigen 43
AIDA: adhesin involved in diffuse adherence
AtaPT: aromatic prenyltransferase
ATscFv: single chain antibody fragment
EstPc: esterase from *Psychrobacter cryohalolentis* K5^T^
PalA: *Pseudomonas sp.* autotransporter lipolytic protein
pBAD: plasmid BrkAutoDisplay
BrkA: *Bordetella* serum-resistance killing protein A
CotA: spore coat protein (laccase)
ECH: enoyl-CoA hydratase
ECH-9: L-3-hydroxyacyl-CoA dehydrogenase
FBS: fetal bovine serum
FITC: fluorescein isothiocyanate
GFP: green fluorescent protein
IgA: immunoglobulin A
INP: ice-nucleation protein
IPTG: isopropyl β-d-1-thiogalactopyranoside
LB: lysogeny Broth medium
LPS: lipopolysaccharide
MisL: protein of membrane insertion and secretion
MCS: multiple cloning site
OmpA: outer membrane protein A
OM: outer membrane protein
PAGE: polyacrylamide gel electrophoresis
PBS: phosphate buffered saline
PCR: polymerase chain reaction
SDS: sodium dodecyl sulfonate
hTNFα: antigen human tumor necrosis factor alpha
